# Methionine-Functionalized Graphene Oxide/Sodium Alginate Bio-Polymer Nanocomposite Hydrogel Beads: Synthesis, Isotherm and Kinetic Studies for an Adsorptive Removal of Fluoroquinolone Antibiotics

**DOI:** 10.3390/nano11030568

**Published:** 2021-02-25

**Authors:** Sushma Yadav, Anupama Asthana, Ajaya Kumar Singh, Rupa Chakraborty, S. Sree Vidya, Ambrish Singh, Sónia A. C. Carabineiro

**Affiliations:** 1 Department of Chemistry, Govt. V.Y.T. PG Autonomous College, Durg 491001, India; sushmabhilai80@gmail.com (S.Y.); anurakeshbhilai@gmail.com (A.A.); roopachakraborty1991@gmail.com (R.C.); 2 Department of Chemistry, Kalyan PG College, Durg 490006, India; vidsan1987@gmail.com; 3 School of Materials Science and Engineering, Southwest Petroleum University, Chengdu 610500, China; drambrishsingh@gmail.com; 4 LAQV-REQUIMTE, Department of Chemistry, NOVA School of Science and Technology, Universidade NOVA de Lisboa, 2829-516 Caparica, Portugal; sonia.carabineiro@fct.unl.pt

**Keywords:** methionine functionalized, graphene oxide, polymer nanocomposite, hydrogel beads, fluoroquinolones antibiotics, adsorption, isotherms, kinetics, thermodynamics

## Abstract

In spite of the growing demand for new antibiotics, in the recent years, the occurrence of fluoroquinolone antibiotics (as a curative agent for urinary tract disorders and respiratory problems) in wastewater have drawn immense attention. Traces of antibiotic left-overs are present in the water system, causing noxious impact on human health and ecological environments, being a global concern. Our present work aims at tackling the major challenge of toxicity caused by antibiotics. This study deals with the efficient adsorption of two commonly used fluoroquinolone (FQ) antibiotics, i.e., Ofloxacin (OFX) and Moxifloxacin (MOX) on spherical hydrogel beads generated from methionine‒functionalized graphene oxide/ sodium alginate polymer (abbreviated Met-GO/SA) from aqueous solutions. The composition, morphology and crystal phase of prepared adsorbents were characterized by X-ray diffraction (XRD), field emission scanning electron microscopy (FE-SEM), Fourier transform infrared spectroscopy (FTIR), high-resolution transmission electron microscopy (HR-TEM) and thermogravimetric analysis/differential thermogravimetry (TGA/DTG). Batch adsorption tests are followed to optimize the conditions required for adsorption process. Both functionalized and non-functionalized adsorbents were compared to understand the influence of several experimental parameters, such as, the solution pH, contact time, adsorbent dosage, temperature and initial concentration of OFX and MOX on adsorption. The obtained results indicated that the functionalized adsorbent (Met-GO/SA) showed a better adsorption efficiency when compared to non-functionalized (GO/SA) adsorbent. Further, the Langmuir isotherm was validated as the best fitting model to describe adsorption equilibrium and pseudo second-order-kinetic model fitted well for both types of adsorbate. The maximum adsorption capacities of Met-GO/SA were 4.11 mg/g for MOX and 3.43 mg/g for OFX. Thermodynamic parameters, i.e., ∆*G*°, ∆*H*° and ∆*S*° were also calculated. It was shown that the overall adsorption process was thermodynamically favorable, spontaneous and exothermic in nature. The adsorbents were successfully regenerated up to four cycles with 0.005 M NaCl solutions. Overall, our work showed that the novel Met-GO/SA nanocomposite could better contribute to the removal of MOX and OFX from the liquid media. The gel beads prepared have adequate features, such as simple handling, eco-friendliness and easy recovery. Hence, polymer gel beads are promising candidates as adsorbents for large-scale water remediation.

## 1. Introduction

Pharmaceutical compounds are major contaminants as they are extensively used and have long-term effects on the aquatic ecosystems [1]. Antibiotics are some examples, being frequently released into the environment. Such powerful life rescuers enter the ecosystem, especially during an epidemic situation, by different routes such as: human or animal wastes, municipal wastewater treatment plants, nursing home wastes, hospital wastewater, and pharmaceutical manufacturing [2,3,4].

The fluoroquinolones (FQs) antibiotics (Enrofloxacin, Moxifloxacin, Ofloxacin, Ciprofloxacin, and many more) are widely used in the treatment of several bacterial infections of humans and animals [5]. Moxifloxacin (MOX) and Ofloxacin (OFX) belong to the FQs antibiotics family. Both drugs are generally applied in veterinary practices and are, medically speaking, very effective against urinary and respiratory infections, caused by Gram-positive and Gram-negative aerobic bacteria [6,7]. About 50–90% of antibiotic drugs are released to the aquatic media via the domestic sewage, coming from human urine and feces, due to incomplete metabolism in human body or from the effluents of these drugs [8]. As a result, they may cause dangerous side effects, like neurological damage resulting in convulsions, the death of microorganisms and enhancement of drug resistance in bacteria [9,10]. Thus, the removal of FQs from effluents is a crucial issue and there is an imperative need to create cost-effective and technologies to remove such antibiotics from wastewater.

At present, various strategies have been successfully used to remove FQs from aquatic environments, such as photodegradation [11], adsorption [12], Fenton oxidation [13] and biodegradation [14]. Among these removal techniques, we prefer adsorption, due to simplicity and advantages, compared to others, such as lower cost, easy operation, and non-existence of highly toxic by-products [15]. In adsorption, the most important aspect is the choice of adsorbent, that should be easily prepared in a cost-effective way, and must be regenerable up to several cycles. Recently, several adsorbents have been used by many researchers for antibiotic removal, such as montmorillonite-biochar composite [16], Fe_3_O_4_@SiO_2_-chitosan/graphene oxide nanocomposite [17], carbon nanotubes/Fe_3_O_4_ magnetic nanocomposite [18] and MnFe_2_O_4_/activated carbon magnetic composite [19].

In the recent years, several adsorbents have been fruitfully applied by photodegradation or adsorption for the removal of fluoroquinolone antibiotics from wastewater. For instance, Tan et al. [20] used molecularly imprinted polymer nanoparticles for the selective removal of OFX antibiotics in aqueous solution. Bekkali et al. [21] studied the comparative sorption and photocatalytic efficiencies of OFX and ciprofloxacin in aqueous solutions supported on ZnO and TiO_2_ catalysts under UV-light irradiation. Nurchi et al. [22] used grape stalk as a potential biomass for sorption of OFX and chrysoidine from wastewater without regeneration studies. On the other hand, Zhu et al. [23] developed graphene oxide/calcium alginate composite fibers via freeze-drying method using calcium chloride as a cross-linking reagent for the removal of tetracycline from water samples.

Hence, we are aiming at a more effective mechanical performance, with enhanced adsorption efficacy, easy separation and improved biodegradability of the adsorbent. Therefore, we have selected methionine-functionalized graphene oxide/sodium alginate polymer (Met-GO/SA) nanocomposite hydrogel beads for antibiotic removal. All the components in this prepared adsorbent have noteworthy properties.

(1) Graphene oxide (GO) is a novel 2D carbon nanomaterial that captured significant interest because of the abundant high-density surface oxygenated functional groups, like hydroxyl, carbonyl, carboxyl and epoxy [24]. GO also exhibits excellent mechanical properties, electron transport properties, structural flexibility, chemical stability and high surface area [25,26]. Furthermore, if GO is modified with an amino acid, there is an increase in its electrical conductivity and regulation of its surface oxygenated groups by partial reduction [27].

(2) Methionine is a sulfur containing amino acid, thus we aim at adding a sulfur and amino functional group in an attempt to combine those with the GO oxygen groups to obtain functionalized GO. Hence, amino acid functionalized GO, as a non-toxic material, provides enhanced water dispersibility and good biocompatibility. Therefore, it can safely be used in aquatic and biological environments [28].

(3) Sodium alginate (SA) is a natural polymer with many advantages, like biocompatibility, low-cost and nontoxicity [29]. It can originate hydrogels in the presence of calcium chloride (Ca^2+^ cations) due to ionic cross-linking capacity. Hence, it was used in the preparation of the composite SA and Met-GO. This new novel hydrogel, a 3D network of hydrophilic polymers, is crosslinked by hydrogen or covalent bonds and van der Waals interactions. This polymer gel does not dissolve in aqueous solution but can absorb large volumes of water. Hydrogels have been applied in drug delivery, pharmaceuticals and thus solve many environmental problems, given their important properties (such as mechanical strength, biocompatibility and not toxicity) [30,31].

This study combines the advantages of SA polymer and functionalized Met-GO materials by cross-linking with calcium chloride. The new polymer nanocomposite hydrogel beads (Met-GO/SA) were applied for adsorbing fluoroquinolone antibiotics (MOX and OFX) from water. The effects of functionalized Met-GO/SA on the structure, morphology and thermal stability were systematically examined by means of X-ray diffraction (XRD), Fourier transform infrared spectroscopy (FTIR), field emission scanning electron microscopy (FE-SEM), high-resolution transmission electron microscopy (HR-TEM) and thermogravimetric analysis/differential thermogravimetry (TGA/DTG). After cross-linking, the resultant gel beads were easily handled and quickly separated from the aqueous solution. The influence of different parameters on MOX and OFX adsorption, like pH, dosage, contact time and initial drug concentration were tested, using functionalized (Met-GO/SA) and non-functionalized (GO/SA) polymer gel beads. Further, the effects of ionic strength and temperature on the adsorption of antibiotics were also investigated. Moreover, the adsorption isotherms, kinetics, thermodynamics and the reusability of Met-GO/SA were also discussed.

## 2. Experimental Segments

### 2.1. Materials and Instruments

Two commonly used FQs antibiotics including Moxifloxacin (MOX, >98%), Ofloxacin (OFX, >99%) were purchased from Sigma Aldrich (Bangalore, India). Graphite powder was purchased from Merck (Mumbai, India). L-methionine (C_5_H_11_NO_2_S) was acquired from Alfa Aesar (Mumbai, India). The chemical includes sulphuric acid (H_2_SO_4_), hydrogen peroxide (H_2_O_2_), potassium permanganate (KMnO_4_), sodium hydroxide (NaOH) and hydrochloric acid (HCl). All chemicals were purchased from Merck (Mumbai, India) and used without prior purification. Solutions were prepared in deionized water. The stock solution of each FQ was individually prepared in a concentration of 100 mg/L in triple deionized water and kept in the cold, dark place for further use. The chemical structures and other properties of the used FQs are presented in Table 1.

The morphologies of synthesized Met-GO/SA nanocomposite were studied by high resolution transmission electron microscopy (HR-TEM, JEOL-JEM 2100, Cochin, India). X-ray diffraction (XRD) diffractograms were obtained on a PANalytical-X’Pert^3^ powder instrument (Raipur, India). The surface functionalities of the sample were recorded by Fourier transform infrared (FTIR) spectroscopy using a Thermo Nicolet Avatar 370 (Cochin, India), in range of 4000–500 cm^−1^. The surface morphology of GO/SA and Met-GO/SA were determined by FE-SEM (Zeiss equipment, Roorkee, India). Thermal stability of the functionalized and non-functionalized adsorbent was tracked by a TGA/DTG analyzer (model EXSTAR TG/DTA 6300, Roorkee, India) using 10 mg of sample, heating from the room temperature to 810 °C, under nitrogen flow, at 10 °C min^−1^. A pH-meter (Systronics-361 model, Bhopal, India) was used to obtain the pH of the solutions. The FQ antibiotics concentrations were followed by ultraviolet-visible (UV-Vis) spectrophotometry (Systronics UV-Vis spectrophometer-117, Bhopal, India).

### 2.2. Synthesis of Met-GO/SA Polymer Nanocomposite Hydrogel Beads and Individual Components

#### 2.2.1. Synthesis of GO

Firstly, GO was obtained from commercial graphite powder by the modified Hummers method [34,35]. Briefly, 1.0 g of graphite was stirred in concentrated H_2_SO_4_ (50 mL) at 0 °C. Then, 4.0 g of KMnO_4_ were added slowly under continuously stirring, maintaining the temperature below 10 °C. The mixture was then continuously stirred for 2 h at 10 °C. The mixture was left alone to naturally heat to room temperature and then stirred for 1 h at 35 °C, and diluted with 50 mL of deionized water (DW), maintaining the temperature below 98 °C. The suspension was stirred for 1 h and diluted with 150 mL of DW, then 20 mL of 30% H_2_O_2_ were added to minimize the residual KMnO_4_ yielding in a yellow-brownish mixture. The mixture was filtered and washed several times with 10% HCl and DW to remove acid residues. The resulting product was dried at 60 °C under vacuum for 24 h.

#### 2.2.2. Synthesis of Met-GO

Amino acid functionalized GO were prepared according to literature [28,36], with slight modifications. In brief, 0.5 g of GO were dispersed in DW (15 mL) and ultrasonicated for 30 min. Then 0.5 mol/L^−1^ L-methionine was added under continuous stirring. Afterwards, the Met-GO dispersions were mixed for 1 h under magnetic stirring and aged for 24 h without stirring at room temperature. The resultant material (Met-GO) was centrifuged (10,000 rpm) and washed 1–2 times with DW to remove untreated amino acids. After each washing, the mixture was centrifuged and decanted. The synthesized Met-GO was oven dried at 60 °C for 12 h and then manually grinded down to a fine powder for further use.

#### 2.2.3. Synthesis of Met-GO/SA Beads

**Figure 1:**
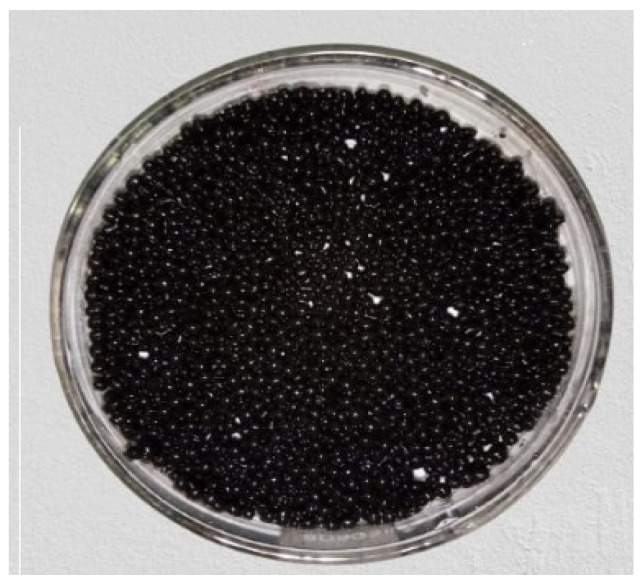
Images of Met-GO/SA polymer nanocomposite hydrogel beads.

Met-GO/SA polymer nanocomposite hydrogel beads were obtained according to [37], with some modifications. 1.5 g of sodium alginate was dispersed into 75 mL deionized water with constant magnetic stirring to obtain a viscous solution. Then 1.0 g of Met-GO nanoparticles were added and stirring continued for 2 h to form a homogeneous solution. Further, the Met-GO/SA solution was then added dropwise into a 0.2 mol L^−1^ CaCl_2_ solution with stirring. The Met-GO/SA polymer nanocomposite hydrogel beads were harden after staying 24 h in the CaCl_2_ solution, to yield stable beads. Finally, the synthesized polymer nanocomposite hydrogel beads were continuously washed with DW to eliminate the excess of CaCl_2_ on the beads surface, and stored in DW for further use. The prepared Met-GO/SA polymer nanocomposite hydrogel beads are shown in Figure 1. A similar procedure was repeated for the synthesis of Graphene oxide/sodium alginate (GO/SA) without the addition of L-methionine.

### 2.3. Adsorption Studies

These tests were performed in batch conditions with a Temp-star water bath shaker incubator. Batch tests are important as there is the need for continuous contact between adsorbent and adsorbate until equilibrium is attained. In a typical experiment, the needed amount of adsorbent was put into a conical flask with 20 mL of MOX or OFX with 20 mg/L concentration separately for both types of adsorbents (Met-GO/SA and GO/SA).

The effects of several operational parameters, like pH, dosage, temperature and drug concentration for both adsorbents (GO/SA and Met-GO/SA) were studied individually and their final adsorption capacity were used for final comparative study. Each parameter was varied separately, while other parameters were kept constant. The reusability cycles were also studied to optimize the adsorption efficiency of Met-GO/SA. To study the effect of pH of the fluoroquinolone antibiotics on the removal capacity, a predetermined contact time, dosage and initial concentration were used. The pH values (2–9 pH) of the antibiotic solutions were adjusted with 0.1 M NaOH and 0.1 M HCl. Further, to optimize the effect of adsorbent dosage in removal efficiency, different amounts of GO/SA (0.10 g to 0.45 g) and Met-GO/SA (0.10 g to 0.35 g) were added to 20 mL of a 20 mg/L concentration of antibiotic solutions. To obtain the adsorption isotherms, solutions of the target antibiotics solutions (optimal pH was 7) at different concentrations (5 to 50 mg/L) were shaken with the adsorbents, at room temperature. Moreover, the kinetic study tests were done using 20 mg/L concentration of MOX or OFX solutions shaken separately with both adsorbents at different durations. The temperature effect was optimized for three different temperatures 35 °C, 45 °C and 55 °C. At the end of equilibrium, the adsorbents and drug solution were easily separated by decantation or filtration processes, since hydrogel beads are hydrophobic in nature, so they are not soluble in water. The remaining concentrations of drug solution were analyzed by UV-vis spectrophotometry at 290 nm for MOX and 288 nm for OFX drugs. The removal rate (*RE_e_*%) and adsorption capacity (*q_e_*) of the Met-GO/SA and GO/SA at the equilibrium stage were obtained by the following Equations (1) and (2):

(1) $$RE_{e}\;\left(\%\right)=\;\frac{C_{0}-\;C_{t}}{C_{0}}\;\times \;100$$

(2) $$q_{e}=\;\frac{\left(C_{0}-C_{t}\right)\;V\;}{m}$$

where *C*_0_ and *C_t_* (mg/L) are respectively, the initial and the equilibrium concentrations of drug solution, *m* is the adsorbent mass (g) and *V* is the volume of solution (L).

Moreover, for statistical purposes, each experiment (of adsorptions for isotherms and kinetics) was conducted in duplicate and the mean values are presented. All those mean values were calculated using Microsoft Office (Professional Plus 2016, Washington, DC, USA).

All data were plotted using linear equations in Origin Pro 8.0 (graphing and analysis software, Northampton, MA, USA). For model fittings, the regression coefficient (*R*^2^) of the linear models was taken into consideration.

## 3. Results and Discussion

### 3.1. Characterization of Met-GO/SA

#### 3.1.1. FTIR Analysis

**Figure 2:**
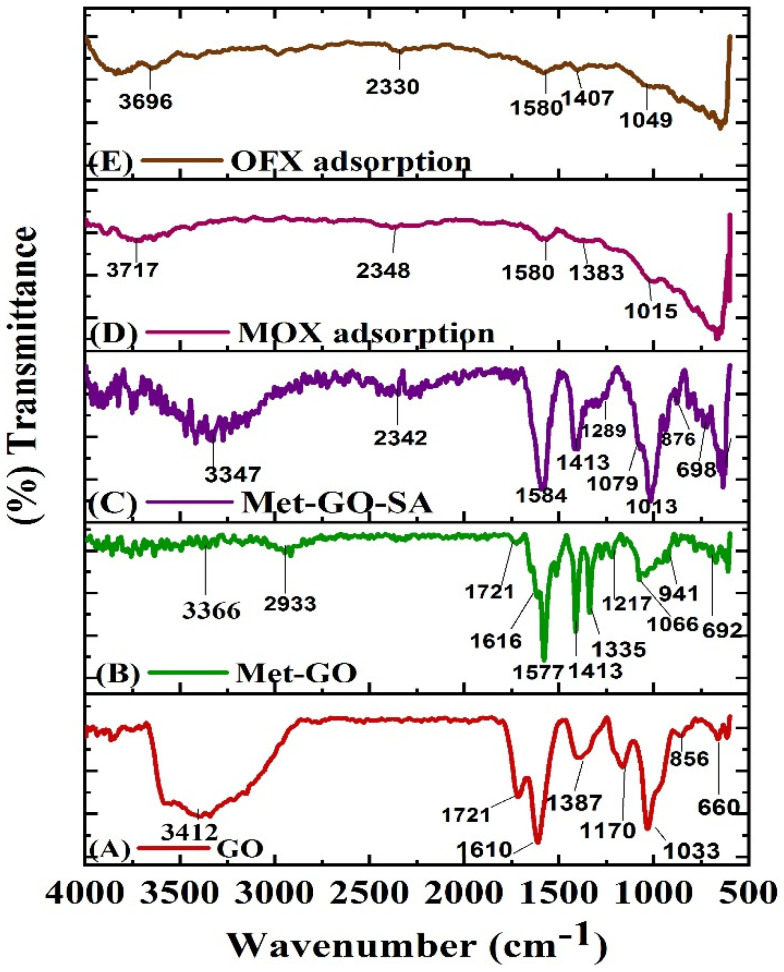
FTIR spectra of (**A**) GO, (**B**) Met-GO and (**C**) Met-GO/SA (**D**) after OFX adsorption (**E**) after MOX adsorption.

The functional groups of the GO, Met-GO and Met-GO/SA were analyzed by FTIR. As shown in Figure 2A, peaks at 3412, 1721, 1610, 1387, 1170, and 1033 cm^−1^ were found in GO. This could be ascribed to the bending and stretching vibrations of O–H, C=O stretching of carboxylates and conjugated carbonyls, aromatic C=C, C–O–C, CO–H and C–O stretching vibration, respectively, and the peaks at 856 and 660 cm^−1^ appear to be due to C–H stretching vibrations [38]. This result shows presence of hydroxyl and oxygen groups on the GO surface. In case of Met-GO (Figure 2B), the existence of a band at 3366 cm^−1^ was due to N–H, O–H, stretching vibrations. A small peak at 2933 cm^−1^, was assigned to the stretching and bending vibration of saturated –CH_2_ bonds [39]. The peak at 1721 cm^−1^ refers to the C=O stretching vibration. In addition, the thiol group (–SH) modification on GO was shown by the existence of peaks around 1413 and 1335 cm^−1^ on Met-GO, attributed to a C–S vibration [40]. C=C, N–H, and C−O stretching vibrations of alkoxy groups from Met-GO, contributed the peaks at 1616 cm^−1^, 1577 cm^−1^ and 1066 cm^−1^, respectively [41]. The bands at 692 cm^−1^ and 1217 cm^−1^ show vibrational C–S bonds and –CH_2_–S wagging, respectively [42]. The peak at 941 cm^−1^ is attributed to the C–O stretching vibration of alcohol in the as-prepared Met-GO. All the characteristic GO peaks are found in Met-GO, with a small shift in wavenumber. As indicated by the chemical modifications, the shift was caused by the GO surface functional groups, formed by shell functionalization. Further, spectral changes were also found for Met-GO/SA nanocomposite (Figure 2C), and a substantial shift of such peaks occurred: (Met-GO) from 3366 cm^−1^ (–OH and –NH) to 3347 cm^−1^, 1577 cm^−1^ (N–H) to 1584 cm^−1^, 1413 cm^−1^ (C–S) to 1407 cm^−1^ and 1066 cm^−1^ (C–O) to 1079 cm^−1^, showing that the introduction of SA was successful. Other bands at 1013, 1289, 876 and 698 cm^−1^ were found in the Met-GO/SA, which could be deformation of C–O stretching vibration, C−H stretching of epoxy groups, C−H stretching and C–S vibration respectively [43]. These changes in FTIR spectra suggest that successful interaction of SA with Met-GO took place leading to surface modification. Moreover, FTIR spectra of Met-GO/SA after antibiotic adsorption are shown in Figure 2D,E. After MOX and OFX adsorption, a significant shift of those peaks (Met-GO/SA) is observed from 3347 cm^−1^ (–OH stretching) to 3717 and 3696 cm^−1^, 2342 cm^−1^ (O=C=O, C–H) to 2348 and 2330 cm^−1^, 1584 cm^−1^ (N–H) to 1580 cm^−1^ and 1413 cm^−1^ (C–S–, S=O) to 1383 and 1407 cm^−1^, 1079 cm^−1^ (C–O) to 1015 and 1049 cm^−1^, for MOX and OFX, respectively. These changes indicate that chemical interactions take place between antibiotics and the functional groups present on the Met-GO/SA adsorbent surface. Hence, it is distinctly proven that the functional groups existing on the Met-GO/SA surface are significantly involved in capturing drug molecules [44].

#### 3.1.2. XRD Analysis

**Figure 3:**
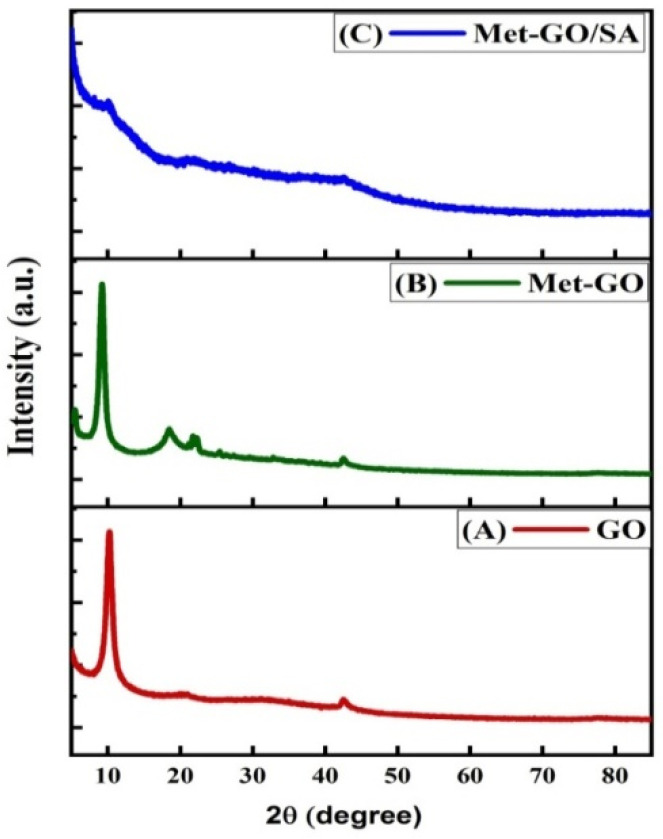
XRD patterns of (**A**) GO, (**B**) Met-GO and (**C**) Met-GO/SA.

GO, methionine functionalized GO and Met-GO/SA powders were analyzed by XRD, to evaluate their crystallinity. Results are shown in Figure 3. For GO (Figure 3A), a sharp peak at 10.26° and other peaks at 20.13° and 42.51°, are ascribed to oxygen-containing functional groups on GO surface [45]. For Met-GO (Figure 3B), there are peak shifts from 10.26° to 9.2° and from 42.51° to 42.48°, with an average interplane distance of 10.71 Å. The shift is ascribed to the enlarged distance between GO inter layers after methionine introduction, due to the addition of sulfur and amine functional groups between the exfoliated GO layers. A new broad peak also appears in the XRD pattern of Met-GO at 18.51° and another at 21.74°. Met-GO/SA (Figure 3C) showed broad peaks at 10.05°, 20.92°, and 42.59°, that correspond to an average interlayer spacing of 10.43 nm. Further, the particle average crystalline size was obtained by the Debye-Scherrer Equation (3) [46]:

(3) $$D=\;\frac{K\lambda }{\beta \cos\theta }$$

where *D* describes the mean diameter of particles, *λ* is the wavelength (0.1541 nm), K is a constant (0.89), *β* is FWHM (the full width at half-maximum) in radians, and θ is the half diffraction angle. According to this equation, particle sizes of GO, Met-GO and Met-GO/SA were estimated to be 3.29 nm, 3.92 nm and 1.19 nm respectively.

Here, the d-spacing was also calculated by the Bragg’s Equation (4) [47]:

(4) nλ=2dsinθ

where *n* is an integer and *d* is the interplanar distance.

The average particle size, FWHM and *d* spacing of GO, Met-GO, Met-GO/SA are summarized in Table 2.

#### 3.1.3. HR-TEM Analysis

**Figure 4:**
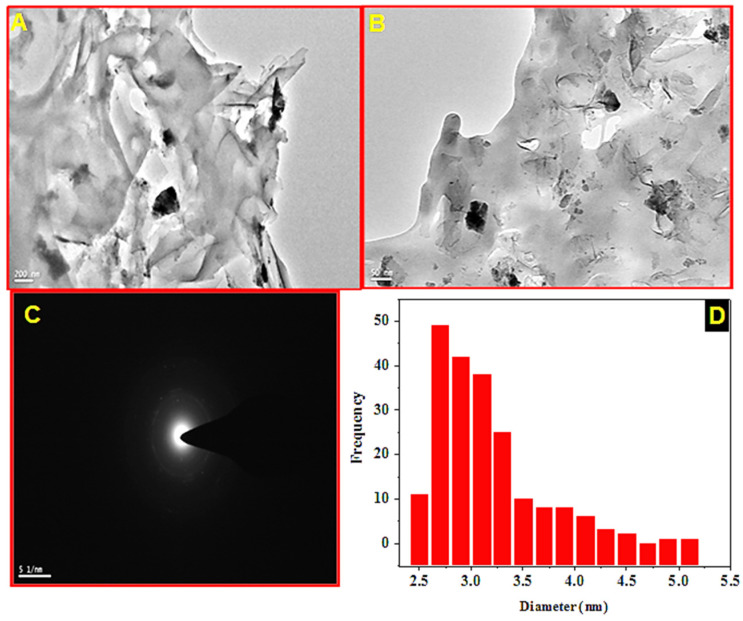
(**A**,**B**) HR-TEM images (with different magnifications) (**C**) SAED pattern and (**D**) size distribution curve of Met-GO/SA.

The morphology of Met-GO/SA was studied by HR-TEM. Figure 4A,B show two representative TEM micrographs with different magnifications. A typical wrinkled sheet-like structure with different sizes of particle agglomeration is seen, that may come from the amino acid functionalization of GO, and partial re-stacking of GO layers [36]. Figure 4C shows a selected area electron diffraction (SAED) pattern of Met-GO/SA, that allows to evaluate sample crystallinity. The diffused ring shown which confirms the sample amorphous nature. The average particle size distribution of Met-GO/SA (Figure 4D) was also determined by Image J (Madison, WI, USA) [48], and the average particle size was 2.91 ± 0.03 nm.

#### 3.1.4. FE-SEM Analysis

**Figure 5:**
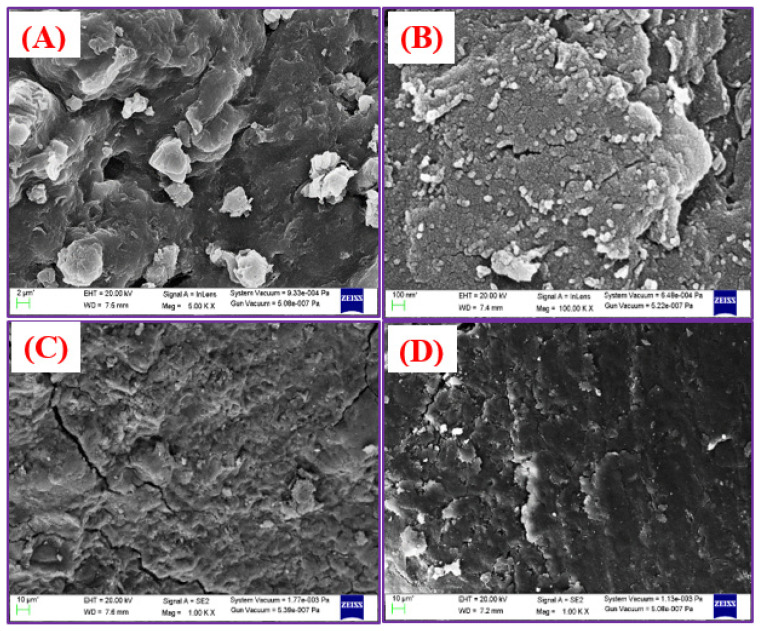
FE-SEM images of (**A**) GO/SA (**B**) Met-GO/SA before adsorption (**C**) after OFX adsorption (**D**) after MOX adsorption on Met-GO/SA surface.

Figure 5A shows the surface morphologies of GO/SA before adsorption. This image demonstrates an irregular surface with high porosity. Functionalized Met-GO/SA is shown in Figure 5B, depicting clear edges and rougher surface before drug adsorption. It may be due to possible aggregation of amino acids that developed micropores, providing more active sites on the adsorbent surface. Subsequently, significant changes were seen on the surfaces after antibiotic adsorption, i.e., they became smoother and a bulky coated intense layer was found, as shown in Figure 5C,D.

#### 3.1.5. TGA/DTG Analysis

**Figure 6:**
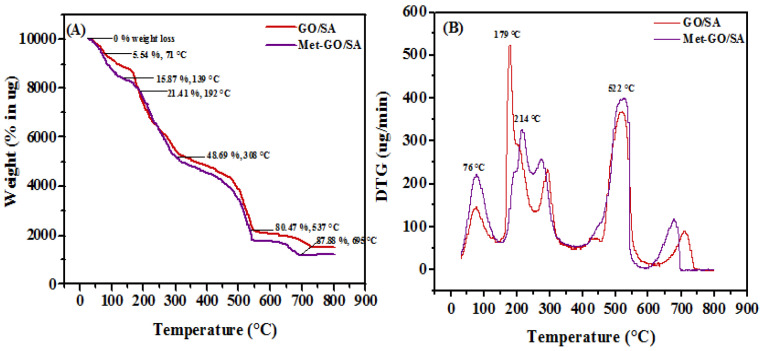
Thermal analysis curve (**A**) TGA (**B**) DTG for GO/SA and Met-GO/SA.

The relative thermal stability of the adsorbents could be assessed by thermogravimetry. The TGA-DTG curves for the GO/SA and Met-GO/SA were measured from room temperature to 810 °C and are shown in Figure 6A,B. It is notable that all the degradation patterns, in four stages, are similar, for both adsorbents. A mass loss of Met-GO/SA in the temperature range of 71 °C to 139 °C is approximately 15.87%, mainly due to the evaporation of physically adsorbed water. The weight loss detected from 192 °C to 308 °C is due to desulfonation and loss of the methionine acid [41]. Above 308 °C, the preliminary degradation of alginate begins, and a loss of weight is observed, thus leading to decomposition of all oxygen-containing functional groups of Met-GO/SA [49]. A large weight loss, i.e., 80.47%, is observed at around 537 °C. After 695 °C, the weight loss (87.88%) curve reaches a constant. Further, DTG curves for both adsorbents show two intense exothermic peaks around 179 °C and 522 °C. The above results indicate that Met-GO/SA has a great thermal stability below 214 °C and the variation of TGA curves also show the modification of GO/SA surface.

### 3.2. Adsorption Tests

Adsorption tests were performed by both functionalized (Met-GO/SA) and nonfunctionalized (GO/SA) adsorbent at different conditions with the variation of adsorption parameters such as time, pH, dosage and concentration. The previously described experimental design (Section 2.3) was followed. Each parameter was varied by individually, while others were kept constant with predetermined conditions, as explained above.

#### 3.2.1. Effect of Solution pH

**Figure 7:**
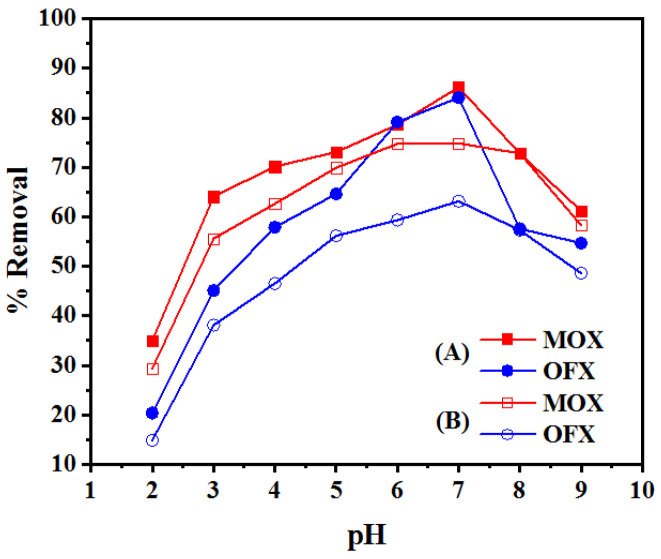
Effect of solution pH on removal rate of MOX and OFX (**A**) by Met-GO/SA (initial concentration: 20 mg/L, dosage: 0.25 g, temperature: 35 °C, time: 140 min) (**B**) by GO/SA (initial concentration: 20 mg/L, dosage: 0.4 g, temperature: 35 °C, time: 200 min).

The effect of pH on FQ adsorption on Met-GO/SA and GO/SA are illustrated in Figure 7A,B, respectively. The observed results for MOX and OFX can be due to pH-dependency and surface charge of the adsorbent. The pKa values for MOX are pKa_1_ = 6.43; pKa_2_ = 10.63 and for OFX pKa_1_ = 6.1 and pKa_2_ = 8.28 [32]. FQ antibiotics have zwitterionic (pH between pKa_1_ and pKa_2_), positively charged (cationic; pH < pKa_1_), negatively charged (anionic; pH > pKa_2_) parts. In this study, FQ act as a zwitterion, with different pKa values. As seen in Figure 7A,B, as the pH increases from 2 to 6, the recovery of FQs increases significantly. However, when pH exceeds 7, a substantial decrease in the removal rate is observed. This was ascribed to deprotonation of the binding sites on FQs and adsorbents [50]. The results at pH 7.0 showed a maximum adsorption of MOX and OFX, which increased with the zwitterionic form increase. At this pH, the Met-GO/SA and GO/SA surfaces had negative charge, whereas MOX and OFX were in zwitterionic form. In such a case, the net charge of FQ antibiotic was zero. Therefore, this indicates that both adsorbents possessed a negatively charged surface, given the large amount of oxygen functional groups and protonated amine groups, which contributed to the adsorption of MOX and OFX. It should be also noted that Met-GO/SA had better adsorption efficiency for MOX and OFX than GO/SA given the negatively charged sulfur containing amino acid on the surface of Met-GO/SA. Further, as seen in Figure 7A,B, adsorption was lower, when the solution pH was acidic because of the decline of the negative charge on the adsorbent surface at low pH leads to repulsion of the positively charged FQs. Such results are also reported in literature [51,52,53]. Here, the optimum pH for maximum adsorption of MOX and OFX was 7. In these cases, FQs can be adsorbed onto the adsorbent surface by π-electron-donor–acceptor (EDA) process, electrostatic interaction and hydrophobic interaction [54].

#### 3.2.2. Effect of Adsorbent Dosage

**Figure 8:**
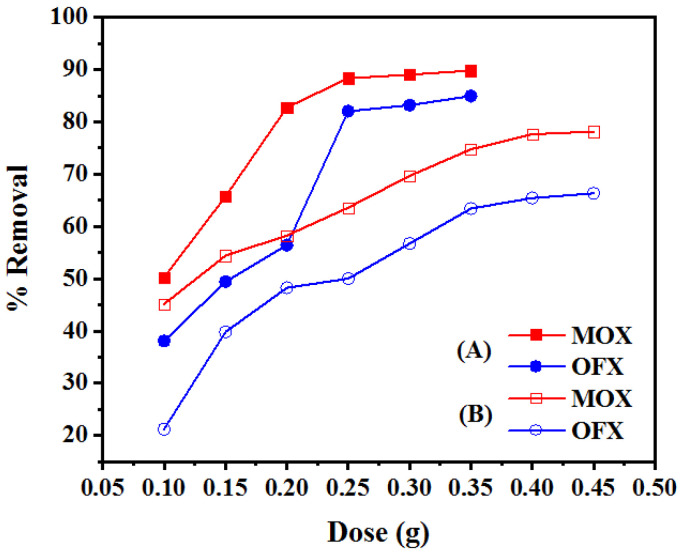
Effects of adsorbent amount on removal rate of MOX and OFX in single-component system onto (**A**) Met-GO/SA (at pH: 7, concentration: 20 mg/L, temperature: 35 °C, time: 140 min) and (**B**) GO/SA (at pH: 7, concentration: 20 mg/L, temperature: 35 °C, time: 200 min).

The adsorbent dosage effect was tested by increasing its amount from 0.1 g to 0.45 g. Figure 8A,B show the dosage effect of Met-GO/SA and GO/SA, on the removal of both antibiotics. As shown in Figure 8A, the removal of MOX increased from 50.26% to 88.33% and of OFX increased from 38.08% to 82.03%. A very slow increase was observed when the adsorbent dosage of Met-GO/SA increased from 0.1 to 0.25 g. Further, in the case of GO/SA (Figure 8B) the removal increased from 45.16% to 77.64% for MOX and from 21.22% to 65.44% for OFX, when the dosages increased from 0.1 to 0.4 g. Increasing the amount of the adsorbent, accelerates the availability of adsorption site. Hence, leads to an initial increased adsorption that later gets retarded due to unavailability, when all active sites are pre-occupied with the adsorbate. The surface charge of the adsorbent has interactions with the adsorbate molecules. Therefore, optimum dosages were chosen as 0.25 g and 0.4 g for Met-GO/SA and GO/SA, respectively, for the adsorbates MOX and OFX. Met-GO/SA showed better removal efficiency compared to GO/SA in the removal of FQ antibiotics.

#### 3.2.3. Effect of Contact Time

**Figure 9:**
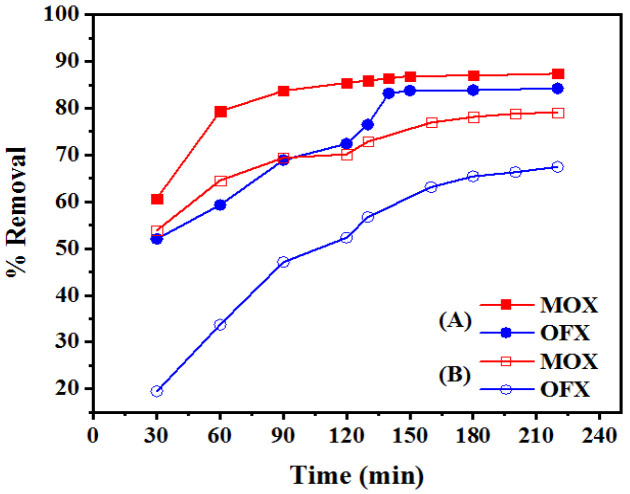
MOX and OFX adsorption on (**A**) Met-GO/SA (pH: 7, concentration: 20 mg/L, dosage: 0.25 g, temperature: 35 °C) and (**B**) GO/SA (pH: 7, concentration: 20 mg/L, dosage: 0.4 g, temperature: 35 °C) as a function of reaction time.

The adsorption of FQ antibiotics by Met-GO/SA and GO/SA was studied by changing the contact time, as shown in Figure 9A,B. Figure 9A shows the influence on the removal of MOX and OFX from 30 to 220 min. The results revealed that up to 140 min, the removal rapidly increased from 60.69% to 86.39% for MOX and 52.07% to 83.19% for OFX. The fast initial adsorption of FQ can be explained by the availability of a large number of active sites on the Met-GO/SA, initially being unoccupied to promote easy adsorption at these active sites. Figure 9B shows the results for GO/SA in the time range of 30–220 min. The result illustrates that up to 200 min, the removal increased from 53.9% to 78.87% for MOX and 19.48% to 68.93% for OFX. Therefore, when comparing the functionalized and non-functionalized materials, Met-GO/SA showed a better performance and the adsorption rate was almost constant after 140 min, indicating that the equilibrium was reached, with high removal rates. For GO/SA, the rate was almost constant after 200 min, but the FQ removed was lower, when the adsorption equilibrium was reached. After that, the adsorption efficiency remained constant. This suggests that the adsorbent surface was saturated and no more adsorbate could be adsorbed. Hence, the optimal contact time was selected as 140 min and 200 min. These values were later used in subsequent experiments using Met-GO/SA and GO/SA as adsorbents.

#### 3.2.4. Effect of Initial Concentration

**Figure 10:**
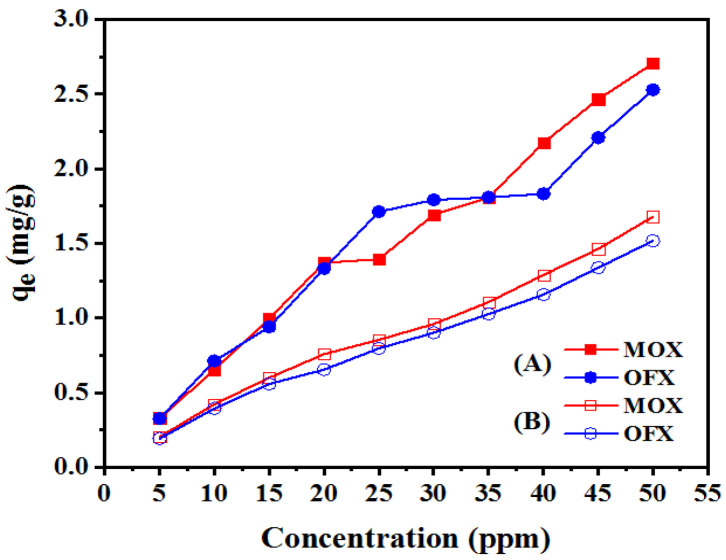
MOX and OFX adsorption on (**A**) Met-GO/SA (Adsorption conditions: time = 140 min; temperature = 35 °C; and adsorbent dosage = 0.25 g) and (**B**) GO/SA (Adsorption conditions: time = 200 min; temperature = 35 °C; and adsorbent dosage = 0.4 g).

The initial concentration of the antibiotic is very important, since; a specific amount of adsorbent can only adsorb a given quantity of adsorbate. Figure 10A,B show the adsorption of FQ by Met-GO/SA and GO/SA, respectively. The results revealed that the adsorption capacity (*q_e_*) increased from 0.32 to 2.70 mg/g for MOX and 0.32 to 2.53 mg/g for OFX upon increasing the concentration as indicated in Figure 10A. However, in case of GO/SA adsorbent the adsorption capacity increased from 0.20 to 1.67 mg/g for MOX and 0.19 to 1.51 for OFX, as represented in Figure 10B. Figure 10A,B show that the adsorption capacity increases with antibiotics concentration increase. It may be due to driving force of mass transfer that increased with the increase in the initial antibiotic concentration, to promote the movement of adsorbate molecules from the bulk solution to the surface of the particle [55]. A similar trend of adsorption was observed using 0.4 g of GO/SA as an adsorbent for different initial concentrations of MOX and OFX. However, the adsorption of MOX and OFX using GO/SA was much lower than using Met-GO/SA. Therefore, we used an initial concentration of 20 mg/L in following equilibrium experiments.

### 3.3. Adsorption Isotherm Studies

**Figure 11:**
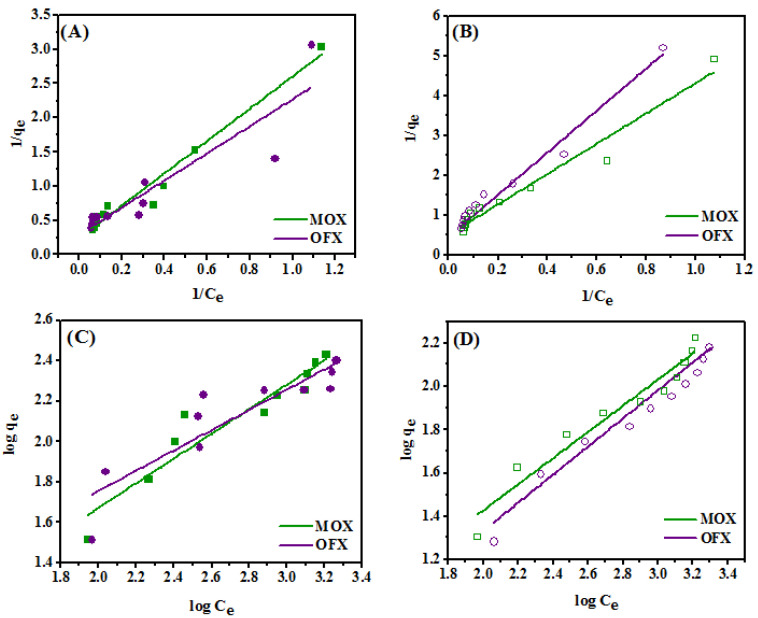
Langmuir models for the adsorption of MOX and OFX onto Met-GO/SA (**A**) and GO/SA (**B**). Freundlich isotherm models for the adsorption of MOX and OFX onto Met-GO/SA (**C**) and GO/SA (**D**). Adsorption conditions for Met-GO/SA as adsorbent (time: 140 min; temperature: 35 °C, adsorbent dosage: 0.25 g, pH: 7) and GO/SA as adsorbent (time: 200 min; temperature: 35 °C; adsorbent dosage: 0.4 g, pH: 7), respectively.

The adsorption isotherm is important to understand the adsorption process. The ability of Met-GO/SA and GO/SA to adsorb MOX and OFX from aqueous solutions in single adsorbate systems was evaluates from the adsorption isotherms. The equilibrium adsorption of the adsorbates (MOX and OFX) onto the Met-GO/SA and GO/SA adsorbent are shown in Figure 11A,B, respectively. The data were fitted to the Langmuir isotherm model. The linear form of this model is expressed in Equation (5) [56] and the results of linear regressions were used to find the best fitting.

(5) $$\frac{1}{q_{e}}=\;\frac{1}{q_{m}}+\;\frac{1}{K_{L}\;q_{m}}\;\times \;\frac{1}{C_{e}}$$

where *C_e_* is the concentration of adsorbate solution at equilibrium (mg/L), *q_e_* is the adsorption capacity at equilibrium (mg/g), *q_m_* (mg/g) is the adsorption capacity and *K_L_* (L/mg) is the Langmuir’s constant. The constants *q_m_* and *K_L_* are obtained by the plot 1/*q_e_* vs. 1/*C_e_*, the intercept and the slope facilitate the calculation of *K_L_* and *q_m_*, respectively.

Further, the adjustment of the adsorption data to the Langmuir model is generally explained using a dimensionless constant separation factor *R_L_*, expressed in Equation (6):

(6) $$R_{L}=\;\frac{1}{1+\;K_{L}C_{0}}$$

where *R_L_* is the separation factor, *K_L_* is the Langmuir constant (L/mg) and *C*_0_ is the initial concentration of antibiotic solution. *R_L_* value provides an idea about the shape of the Langmuir isotherm and the nature of the adsorption [57]. *R_L_* > 1 means an unfavorable monolayer adsorption process, *R_L_* = 1 (linear) means it is favorable (if 0 < *R_L_* < 1), *R_L_* = 0 means irreversible. In this study, the obtain *R_L_* values indicate favorable antibiotic adsorption on both adsorbents.

The Freundlich isotherm consists of an empirical equation dealing with adsorption on a heterogeneous surface. It is expressed in Equation (7) [58]:

(7) $$\log q_{e}=\log K_{F}+\;\left(\frac{1}{n}\right)\log C_{e}$$

where *q_e_* means the adsorption capacity (mg/g), *K_F_* is a constant dealing with the relative adsorption capacity of the adsorbent ((mg/g)(mg/L)*^n^*), *C_e_* is the concentration of solute in the bulk solution at equilibrium (mg/L), and *n* is a constant related with the intensity of adsorption. The constants *K_F_* and *n* can be obtained from the intercept and slope of log *q_e_* vs. log *C_e_* plot.

The data of the adsorption isotherm was fitted by Langmuir and Freundlich isotherm models. The obtained parameters are summarized in Table 3. The Freundlich model confirmed the sorption of antibiotics from aqueous solutions using Met-GO/SA and GO/SA as seen in Figure 11C,D. The values of n (Table 3), between ~1.5 to ~2, confirm the heterogeneity condition (as 1 < *n* < 10). The process is favorable since 1/*n* < 1 [59]. Furthermore, the *R*^2^ values of the Langmuir isotherms are higher than those of the Freundlich model for Met-GO/SA and GO/SA. Thus, the Langmuir isotherm fits better the adsorption data, indicating that the bulk solution adsorbate molecules are adsorbed onto a homogenous monolayer. Therefore, the maximum adsorption capacities of MOX and OFX on Met-GO/SA are 4.115 mg/g and 3.436 mg/g, and are higher than those of GO/SA (2.00 mg/g, 1.798 mg/g, respectively). These values are higher than those of other adsorbents reported in literature, used for FQs, as seen in Section 3.7.

### 3.4. Adsorption Kinetic Studies

**Figure 12:**
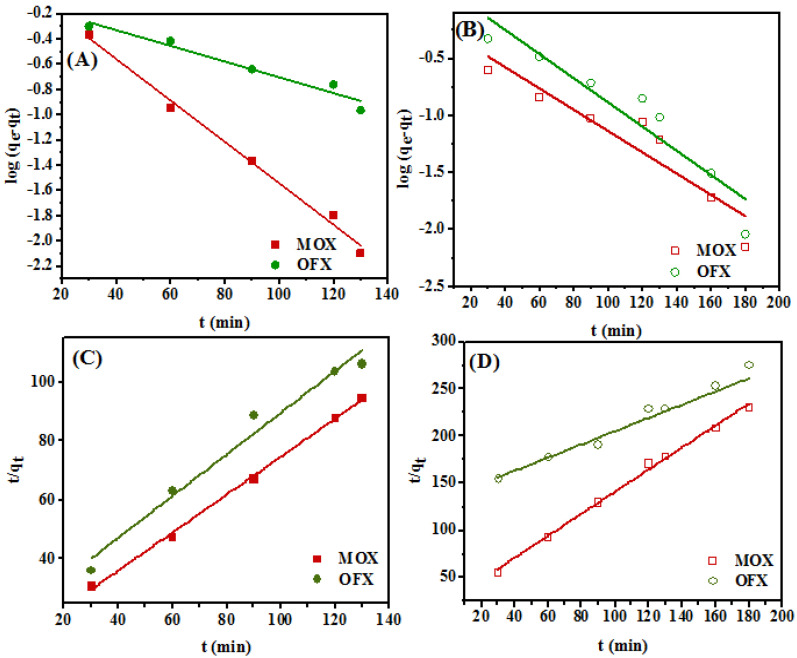
Pseudo-first order and pseudo-second order kinetic models for adsorption of MOX and OFX onto (**A**,**C**) Met-GO/SA as adsorbent (adsorption conditions: concentration = 20 mg/L; temperature = 35 °C; adsorbent dosage = 0.25 g, pH = 7) and (**B**,**D**) GO/SA as adsorbent (adsorption conditions: concentration = 20 mg/L; temperature = 35 °C; adsorbent dosage = 0.4 g, pH = 7), respectively.

Adsorption kinetic studies provide useful data regarding the efficiency of the process and the relationship between the adsorption capacity and time. The adsorption rate primarily depends on the contact time between the liquid and solid as diffusion takes place. Pseudo-first order [60], pseudo-second order [61], and intraparticle diffusion [62] models were used here to find the rate of kinetics and adsorption process. The linear form of the pseudo-first order kinetic rate model can be defined by Equation (8):

(8) $$\log\;\left(q_{e}-q_{t}\right)=\log q_{e}-\;\frac{k_{1}}{2.303}\times t$$

where *q_e_* and *q_t_* are the adsorption capacities of the adsorbent (mg/g), at equilibrium and time *t*, respectively, and *k*_1_ is the pseudo-first order rate constant (min^−1^). The linear graphs of log (*q_e_* − *q_t_*) vs. *t* are represented in Figure 12A,B for Met/GO/SA and GO/SA, respectively. The calculated values of kinetic parameters for all adsorption kinetic models are presented in Table 4. The linear forms of the pseudo-second order rate model and intraparticle diffusion can be expressed by Equations (9) and (10), respectively.

(9) $$\frac{1}{q_{t}}\;=\;\frac{1}{k_{2}q^{2}e}\;+\;\frac{1}{q_{e}}\;\times t$$

where *q_t_* (mg/g) and *q_e_* (mg/g) are the adsorption capacities of the adsorbent, at time *t* and at equilibrium, respectively, and *k*_2_ (g/mg/min) is the pseudo-second order rate constant. Here, *k*_2_ and *q_e_* values can be obtained from the intercept and slope of the plot of *t*/*q_t_* vs. *t*, respectively, and are shown in Figure 12C (for Met/GO/SA) and Figure 12D (for GO/SA). As seen in Table 4, the experimental data revealed a better agreement with the pseudo-second order model, due to the higher correlation coefficient (*R*^2^) values obtained, than with the pseudo-first order model. For both, the adsorbent (calculated) *q_e,(cal)_* values were closer to the experimental *q_e,(exp)_* ones. Hence, it can be assumed that the FQs adsorption on Met/GO/SA and GO/SA follows pseudo-second order kinetics, and chemisorption involving valence forces is taking place at some specific active sites [63]. Moreover, compared with Met/GO/SA and GO/SA, the Met/GO/SA showed a higher equilibrium adsorption capacity (*q_e,(cal)_*) and shorter time to reach adsorption equilibrium, indicating that the amino acid modification process could enhance the affinity towards the antibiotic molecules (Table 4).

**Figure 13:**
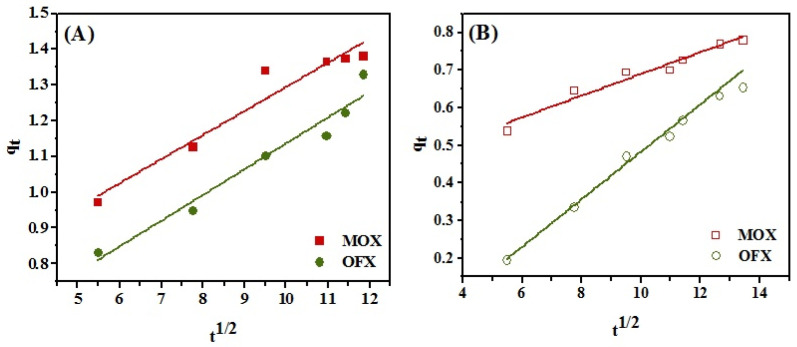
Intraparticle diffusion models for MOX and OFX adsorption onto (**A**) functionalized (Met-GO/SA) (**B**) non-functionalized (GO/SA) adsorbent.

Further, to investigate the possibility of intra-particle diffusion, Morris Weber model (Equation 10) was used, where the linear plots of *q_t_* vs. *t*_1/2_ are displayed in Figure 13A,B for Met/GO/SA and GO/SA, respectively.

(10) $$q_{t}=\;k_{id}{\left(t\right)}^{1/2}\;+C$$

where *q_t_* is adsorption capacity of MOX and OFX in (mg/g), at a given time *t*, *k_id_* is the intraparticle diffusion rate constant (mg/g min^1/2^) and *C* is an intercept which represents the thickness of the boundary layer. Table 4 shows the values of *k_id_* and *C*.

As can be seen in Table 4, for Met-GO/SA, high values of C were found for MOX and OFX. This indicates that the surface adsorption has a large influence in the rate-controlling step [64]. The linear part of the plot for both antibiotics does not pass by the origin showing that intraparticle diffusion is not the sole rate governing factor. Thus, most likely surface adsorption, along with complex sorption mechanism and intraparticle diffusion take place simultaneously [65].

### 3.5. Adsorption Thermodynamics Studies

**Figure 14:**
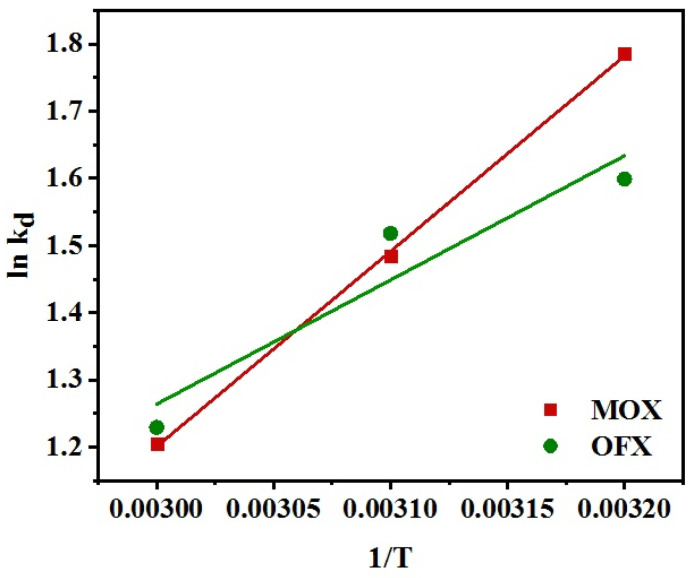
Van’t Hoff plots for the uptake of MOX and OFX on the Met-GO/SA (Adsorption conditions: *C*_0_ = 20 mg/L; time = 140 min; and dosage = 0.25 g at pH = 7).

Thermodynamics studies can demonstrate the adsorption process of MOX and OFX on Met-GO/SA in terms of energy change. The temperature influence on the adsorption of FQ and the thermodynamic parameters were determined by the adsorption experiments at different temperatures from 35 to 55 °C. The Gibbs free energy (Δ*G*°), entropy (Δ*S*°) and enthalpy (Δ*H*°) for the adsorption system were calculated using Equations (11)–(13) [66] and the results are shown in Table 5.

(11) $$\Delta G^{{}^{\circ}}\;=\;\Delta H^{{}^{\circ}}-T\Delta S^{{}^{\circ}}$$

(12) $$\Delta G^{{}^{\circ}}=\;-RTlnk_{d}$$

(13) $$lnk_{d}=\;\frac{\Delta S^{{}^{\circ}}}{R}-\;\frac{\Delta H^{{}^{\circ}}}{RT}$$

where *R* is universal gas constant (8.314 J/mol K), Δ*H*° is the enthalpy change (kJ/mol), Δ*G*° is Gibbs free energy change in a given process (kJ/mol), Δ*S*° is entropy change (J/mol/K), *k_d_* is the thermodynamic equilibrium constant representing adsorbate ion distribution (mL/g) and *T* is the absolute temperature (in K). Further, the Van’t Hoff plot of ln*k_d_* vs. 1/*T* for the Met-GO/SA adsorbents shows a straight line for both antibiotics (Figure 14). These expressions were used to calculate the Δ*H*° and Δ*S*° values using the slope and intercept, respectively. As presented in Table 5 a continuous decrease in Δ*G*° values showed the viability and spontaneous nature of the adsorption process. The negative values of Δ*H*° for MOX and OFX adsorption by Met-GO/SA were −24.19 and −15.38 kJ/mol, respectively, showing the antibiotic binding is an exothermic process [67]. This indicated that the amount removed of both the antibiotics decreased with temperature increase. Moreover, the negative values of Δ*S*° suggested that a decreased randomness at the solid–liquid interface takes place during adsorption of antibiotics on Met-GO/SA [68]. Similar results were also obtained in a previous work for OFX adsorption onto the activated carbon derived from luffa sponge [69], showing negative values of Δ*G*°, Δ*H*° and Δ*S*°.

### 3.6. Effect of Ionic Strength of Solution

**Figure 15:**
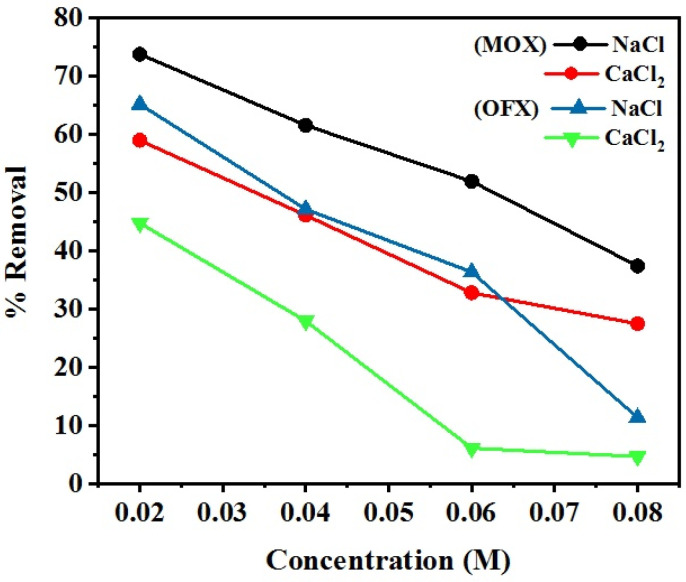
Effect of the ionic strength on adsorption of MOX and OFX on Met-GO/SA.

A comparative study was done to find out the effect of salt (NaCl and CaCl_2_) on FQ antibiotics (MOX and OFX) removal, at an ionic strength that ranging from 0.02 to 0.08 M. As shown in Figure 15, increased salt concentrations in the solution decreased the removal of MOX and OFX by Met-GO/SA adsorbent. A high concentration of NaCl salt (0.08 M) and CaCl_2_ salt (0.08 M) largely decreased the adsorption to 37.40% and 27.46%, respectively, for MOX adsorption. Similarly, the removal of OFX decreased for higher concentrations of NaCl (0.08 M) and CaCl_2_ (0.08 M) with 11.34% and 4.63% adsorption capabilities, respectively. Thus, it can be concluded that different concentrations of salts have negative effects on the electrostatic interactions between adsorbent and adsorbate, caused by the electrostatic screening effect [70]. In addition, CaCl_2_ shows a stronger adsorption inhibition to MOX and OFX than NaCl. This might be because Ca can easily complex with the adsorbent surface groups, that are also the main active sites for antibiotics adsorption. The present result was also confirmed by other similar reports [71,72].

### 3.7. Comparison with Other Adsorbents for FQs Antibiotics Removal

Many adsorbents have been reported for the adsorption of FQs [73,74,75,76,77,78,79,80]. As seen in Table 6, the adsorbent reported in this work had a good adsorption capacity and is advantageous in terms of easy separation. In addition, the binding ability was further enhanced after the functionalization of GO, resulting in a larger adsorption capacity (4.115 and 3.436) mg/g for MOX and OFX, respectively, for the Met-GO/SA adsorbent. This implies that Met-GO/SA has larger potential to be used as adsorbent in the removal of FQ antibiotics than GO/SA.

### 3.8. Regeneration and Reusability of Met-GO/SA

**Figure 16:**
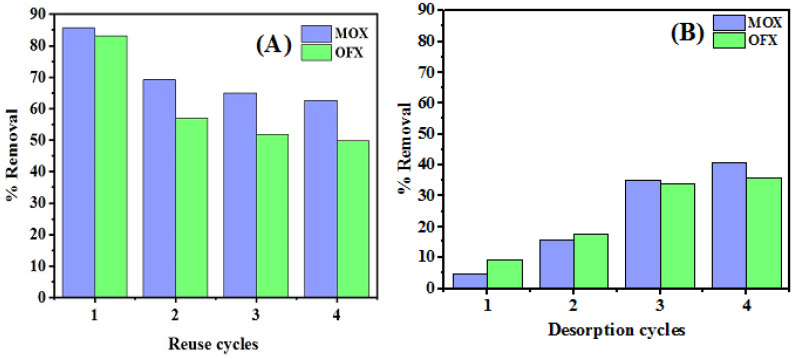
Performance of Met-GO/SA adsorbent through multiple regeneration cycles (**A**) and Desorption cycles (**B**).

In order to determine the potentials of Met-GO/SA adsorbent for practical applications, its reusability was tested. The effects of four consecutive adsorption–desorption cycles were considered, and the results were graphically represented as shown in Figure 16A,B. The amount of adsorbent used for the first cycle was reused in consecutive cycles. The desorption of MOX and OFX using Met-GO/SA was demonstrated with 0.005 N NaCl (20 mL) as desorbing agent. As the Met-GO/SA polymer gel beads were obtained by chelation between Met-GO and CaCl_2_, the bonds were transient and could be described as a reversible or some ion exchange reaction. It was found that if a higher concentration of NaCl, like 0.01 M, was used for desorption, then more Na^+^ ions would replace the Ca^2+^ ions, affecting the crosslinking or disrupting the gel structure of the polymer composite. Moreover, higher concentrations of NaCl lead to more desorption % of antibiotic. In that case, the synthesized material was not able to achieve more than two regeneration cycles. In order to achieve more cycles, a lower concentration had to be used and it was found that 0.005 M NaCl was a suitable value.

After each cycle of adsorption, the adsorbent was removed from the solution and washed with deionized water. The results revealed that the removal percentage decreased from 85.65 to 62.63% for MOX and 83.19 to 49.74% for OFX, after 4 cycles as shown in Figure 16A. The desorption process was also repeated for 4 cycles. Desorption percentage (%) removal for MOX and OFX were calculated using the Equation (14) [82]:

(14) $$R\left(\%\right)=\;\frac{D_{t}}{C_{0}-C_{e}}\times 100$$

where *C_0_* and *C_e_* are initial and final concentration of antibiotic solution in mg/L, *D_t_* is the concentration of antibiotic solution (MOX and OFX) in mg/L.

Finally, after four cycles of desorption, 40.53% of the MOX and 35.58% of the OFX were desorbed, as shown in Figure 16B. Therefore, NaCl behaves as a good desorbing agent for FQ antibiotics removal onto Met-GO/SA adsorbent surface, because it is an ionic compound where Na^+^ can bond with drug molecules to make a complex, while Cl^−^ can replace them and bond with the adsorbing sites on the adsorbent, and would desorb the drug molecule from the solution [83]. After several rounds, Na^+^ ion concentration affects the Met-GO/SA adsorbent surface, hence, can significantly disrupt the gel structures [15]. Thus, we can conclude that Met-GO/SA can be effectively used for cleaning the antibiotics contaminated water, showing good adsorption-desorption capacity, without any loss of adsorbent.

## 4. Conclusions

In this work, a functionalized Met-GO/SA nanocomposite was synthesized and efficiently used for the effective removal of commonly used FQ antibiotics (MOX and OFX) from an aqueous solution in single adsorbate systems. Characterization was made by XRD, FTIR and HR-TEM analytical techniques. A batch adsorption system was used and the results showed that the adsorption efficiency depended on various adsorption parameters, such as pH, time, dosage, etc. Additionally, comparative studies on non-functionalized GO/SA were also done for the removal of the MOX and OFX. The adsorption equilibrium of MOX and OFX was better described by the Langmuir isotherm, while the obtained kinetic data followed a pseudo-second order model. The obtained results indicated that a monolayer adsorption of antibiotics occurred on the surface of Met-GO/SA, with the calculated maximum adsorption amounts of 4.115 mg/g for MOX and 3.436 mg/g for OFX. Adsorption thermodynamic studies showed that the adsorption of antibiotics on Met-GO/SA was exothermic and spontaneous. The results of this study indicated that the Met-GO/SA nanocomposite exhibited higher adsorption capacity for MOX and OFX than the GO/SA, in terms of dosage, time and environmental prospects. Therefore, this indicates that the synthesized Met-GO/SA has a remarkable potential as effective adsorbent for removing fluoroquinolone antibiotics (MOX and OFX) from aqueous solutions. Moreover, Met-GO/SA gel beads are easily separable from aqueous solution for regeneration and reusability, without any mass loss.

## Figures and Tables

**Table 1:** Chemical structure and physicochemical properties of FQs antibiotics.

FQ Antibiotic	Structure	Formula	Weight (g mol^−1^)	*λ_max_* (nm)	Ref.
Moxifloxacin	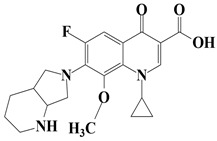	C_21_H_24_FN_3_O_4_	401.431	290	[32]
Ofloxacin	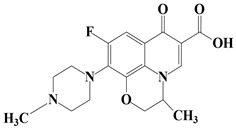	C_18_H_20_FN_3_O_4_	361.368	288	[33]

**Table 2:** Average crystallite size of GO, Met-GO and Met-GO/SA calculated using Debye-Scherrer formula.

Materials	2θ (°)	FWHM	*d*-Spacing (Å)	Size (nm)
GO	10.26 20.13 42.51	1.15 7.27 5.02	10.40	3.29
Met-GO	9.21 18.51 21.74 42.48	0.92 4.98 3.91 2.38	10.71	3.92
Met-GO/SA	10.05 20.92 42.59	7.13 6.46 6.38	10.43	1.19

**Table 3:** Langmuir and Freundlich isotherm parameters for MOX and OFX adsorption on functionalized (Met-GO/SA) and non-functionalized (GO/SA) adsorbents at 35 °C.

Isotherm Models		Moxifloxacin (MOX)		Ofloxacin (OFX)	
		GO/SA	Met-GO/SA	GO/SA	Met-GO/SA
Langmuir model	*q_m_* (mg/g)	2.00	4.115	1.798	3.436
	*K_L_* (L/mg)	0.157	0.101	0.108	0.147
	*R_L_*	0.241	0.331	0.316	0.253
	*R* ^2^	0.963	0.965	0.979	0.827
Freundlich model	*K_F_* (mg/g)(mg/L)*^n^*	1.640	2.844	1.132	5.675
	*n*	1.655	1.644	1.589	2.00
	*R* ^2^	0.947	0.905	0.967	0.754

**Table 4:** Fitting parameters for MOX and OFX adsorption on functionalized (Met-GO/SA) and non-functionalized (GO/SA) using pseudo-first order, pseudo-second order and intraparticle diffusion models.

Kinetic Models	Parameters	Moxifloxacin (MOX)		Ofloxacin (OFX)	
		GO/SA	Met-GO/SA	GO/SA	Met-GO/SA
Pseudo-first order	*k*_1_	0.021	0.037	0.024	0.014
	*q_e_* (mg/g)	1.595	1.208	1.493	1.203
	*R* ^2^	0.879	0.992	0.893	0.954
Pseudo-second order	*k*_2_	0.057	0.040	0.004	0.026
	*q_e_* (mg/g)	0.854	1.552	1.254	1.414
	*R* ^2^	0.996	0.998	0.986	0.977
Intraparticle diffusion model	*k_id_* (mg/g/min)	0.028	0.067	0.058	0.072
	*C*	0.400	0.620	0.115	0.415
	*R* ^2^	0.962	0.942	0.990	0.954

**Table 5:** Thermodynamic parameters of MOX and OFX adsorption onto Met-GO/SA at different temperatures (308–328 K).

Adsorbate	Δ*H*° (kJ/mol)	Δ*S*° (J/mol/K)	Δ*G*° (kJ/mol)		
			308 K	318 K	328 K
MOX	−24.19	−62.59	−4.573	−3.923	−3.283
OFX	−15.38	−35.63	−4.094	−4.013	−3.351

**Table 6:** Comparison of maximum adsorption capacity (*q_max_*) of FQ antibiotics reported for different adsorbents.

Adsorbent	FQ Antibiotic	*q_max_* (mg/g)	Reference
Natural Clinoptilolite Clinoptilolite H-Form	Moxifloxacin Moxifloxacin	1.72 2.71	[73]
Activated carbon nanoparticles (AC)	Tetracycline	1.98	[74]
AC(PPZ)KOH	Ciprofloxacin	2.353	[75]
Modified coal fly ash	Ciprofloxacin	1.547	[76]
MIPs	Norfloxacin	2.99	[77]
Nano- Hydroxyapatite	Norfloxacin Ciprofloxacin	1.486 1.271	[78]
Fe_3_O_4_/CD/AC/SA	Norfloxacin Ciprofloxacin	2.551 3.125	[79]
Spent black tea leaves (SBTL)	Ofloxacin	−3.356	[80]
Nonporous SiO_2_	Ofloxacin	2.1	[81]
GO/SA	Moxifloxacin Ofloxacin	2.0 1.798	This study
Met-GO/SA	Moxifloxacin Ofloxacin	4.115 3.436	This study

## Data Availability

Data will be provided upon request.
